# Well-Preserved Urinary Bladder Anatomy in Rats After Minimally Invasive Surgery

**DOI:** 10.3390/biomedicines13020285

**Published:** 2025-01-24

**Authors:** Daniela Giaquinto, Antonio Palladino, Annunziata Cummaro, Elena De Felice, Vincenzo Esposito, Rosalba Moretta, Sigismondo Castaldo, Eva Di Maro, Paolo de Girolamo, Livia D’Angelo, Chiara Attanasio

**Affiliations:** 1 Department of Veterinary Medicine and Animal Production, University of Naples Federico II, I-80137 Napoli, Italy; daniela.giaquinto@unina.it (D.G.);; 2 Department of Agricultural Science, University of Naples Federico II, I-80055 Napoli, Italy; antonio.palladino@unina.it; 3 Materias Srl, I-80122 Napoli, Italy; 4 Sanidrink Srl, I-80121 Napoli, Italy; 5 School of Biosciences and Veterinary Medicine, University of Camerino, I-62032 Camerino, Italy; elena.defelice@unicam.it; 6 U.O.C. Ricerca, Formazione & Cooperazione Internazionale-A.O.R.N.A. Cardarelli, I-80131 Napoli, Italy

**Keywords:** urinary bladder microarchitecture, animal models, laboratory animals, catheters, antimicrobial coating, antibiotic resistance, medical device

## Abstract

**Background:** The setup of experimental protocols able to preserve the anatomical integrity also in terms of organ microarchitecture is mandatory to ensure result translatability. Also, the maintenance of structural integrity perfectly aligns with the refinement implementation aiming to reduce procedure severity, a key issue in animal studies deemed compulsory from both ethical and legal standpoints. Here we report a detailed description of all peri-operative and post-operative care and clinical evaluation in a surgical rat model to test the efficacy of a catheter functionalized by a peptide coating with antimicrobial and antibiofilm properties, whose efficacy was previously tested in vitro. **Methods:** We used male and female adult Fischer 344 rats (tot *n* = 44, *n* = 22 each sex), which were divided into four experimental groups. Each animal underwent refined surgery for the implantation of a functionalized or standard catheter, depending on the group, and was observed for 7 and 14 days. The surgical refinement strategy was based on the placement of the catheter into the bladder lumen rather than in the urethra. Still in the refinement perspective, ultrasound examination of the bladder was conducted to confirm the in situ position of the medical device at an intermediate time point, 4 or 10 days post-surgery depending on the group, while, at the same time, but also at days 0, 7, or 14 post-surgery, an ultrasound-guided cystocentesis was performed to collect sterile urine. The imaging approach was used in place of metabolic cages to minimize distress to the animals and to ensure reliable and unbiased scientific outcomes. Hematological and biochemical parameters were monitored along the preclinical trial; namely, blood sampling was performed at the beginning (day 0) and at the end of the trial (day 7 or 14 post-surgery depending on the group). **Results:** Clinical scores and biochemical analyses of all animals did not reveal signs of distress or disease. At the endpoints, histological analyses of urinary bladder displayed a well-preserved anatomical structure of the organ without significant signs of inflammatory infiltration into the urothelium. **Conclusions:** Our model represents a refined strategy to achieve the scientific goals required by the preclinical setting related to catheter-associated urinary tract infections. In particular, it ensures the preservation of bladder morphology and urothelium microarchitecture, maintaining an adequate level of animal health and welfare while monitoring the onset of urinary tract infections through the sterile collection of urine in long-lasting experiments.

## 1. Introduction

In animal studies devoted to testing biomedical devices, a crucial aspect to guarantee translatable outcomes from the preclinical setting to the clinical arena is the correct setup of standardized surgical protocols focused on the preservation of organ morphology and tissue microarchitecture. This latter, indeed, being the basis of the maintenance of mechanical resistance, elasticity, and impermeability, is crucial to allow urine storage and therefore the barrier function of the bladder as well as its voiding [1]. From the translational standpoint, this approach is even a major asset in long-lasting experiments, including organs that are particularly exposed to infections in view of their physiological function.

Further, these aspects perfectly align with the refining requirements aiming to reduce the severity of a procedure, a focal point in animal studies from both ethical and legal standpoints [2].

The application and implementation of the refinement principle when using live animals for scientific purposes is mandatory according to the Directive 2010/63/EU, which regulates the protection of animals used for scientific purposes. Therefore, the design of any preclinical study must be systematically underlined by a properly implemented and reviewed refinement strategy [3]. This latter includes the reduction of the severity of the procedures and, in turn, improved animal welfare [3]. Refinement is not addressed to just avoid or minimize adverse effects that may impact any time the life of laboratory animals but covers several aspects of their life, including housing, husbandry and care, technical aspects, periprocedural care, health and welfare monitoring, as well as experimental design and the use of advanced techniques [3,4]. Noteworthy, many preclinical models reported in the literature omit the description of the refinement strategies applied during the experimental procedures. Furthermore, adoption of a new surgical procedure or a variant of an existing one requires extensive knowledge of anatomy, which is relevant to the specific surgical model [5].

The functionalization is based on antimicrobial peptides (AMPs) coating the catheter surface. CAUTIs account for 75% of the total urinary infections, placing an enormous burden on the health care system, as well as being burdensome from an economic standpoint [6,7]. In the clinical setting, the management of CAUTIs has become complicated due to increasing rates of antimicrobial resistance [7,8]. In this scenario, testing the in vivo efficiency of functionalized catheters whose antimicrobial activity has previously been tested in vitro represents a valuable step forward in the prevention of CAUTIs [9,10]. This report outlines a refined approach of a rat surgical model of urinary bladder catheterization to test the efficacy of a functionalized urinary catheter as a possible therapeutic method to prevent catheter-associated urinary tract infections (CAUTIs), drastically limiting the use of antibiotics [11]. The peptide used for catheter functionalization, named Rilk1-cat, consists of a highly compact sequence of just 10 amino acid residues, offering a significant economic advantage by minimizing synthesis and purification costs. Rilk1-cat is of particular interest due to its potential bactericidal activity against a wide range of Gram-positive and Gram-negative strains, including *Salmonella typhimurium*, *Escherichia coli*, and *Staphylococcus aureus*. Additionally, the peptide also shows moderate activity against some yeasts and molds, such as *Candida albicans* and *Aspergillus brasiliensis*. A key advantage of Rilk1-cat is its environmental stability, as it preserves its secondary structure across varying pH conditions and temperatures. These characteristics enable the peptide to retain its antimicrobial efficacy under varying environmental conditions, expanding its potential applications in both clinical and industrial fields.

Among models of catheterization available in the literature, namely surgical, non-surgical, and complete urinary catheterization [11,12,13,14,15], the most appropriate surgical model for our experimental design has been considered while the rat has been selected as the model species. To achieve the research objectives, in terms of animal welfare and data reproducibility, both males and females have been included to ensure a generalizable estimate of the intervention effect across both sexes. Also striving to reduce any environmental variable, the study was compliant with the ARRIVE (Animal Research: Reporting of In Vivo Experiments) guidelines [16].

## 2. Methods

### 2.1. In Vitro Testing

The short antimicrobial peptide sequence (AMP) (under patent) was tested in vitro before functionalization of the device. Cytotoxicity testing was performed according to the method described in the UNI EN ISO 10993-5 and 10993-12 standards [17,18]. The test was carried out on murine fibroblasts (Balb/3T3 cells, clone A31-ATCC CCL-163), cultured in Dulbecco’s Modified Eagle Medium containing 10% CS and antibiotics. Cells were seeded in 96-well plates and allowed to grow for 24 h at 37 °C with 5% CO_2_. The medium was then replaced with the eluted product obtained from both untreated and treated catheters and further diluted in a 1:2 ratio. The experiment was conducted in triplicates.

**Figure 1:**
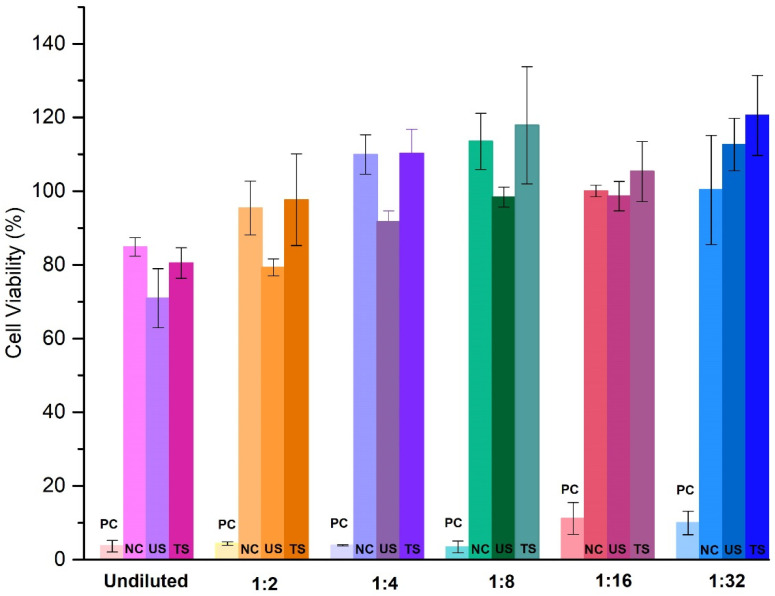
Cell viability expression after treatment with increasing amounts of positive control (PC), negative control (NC), untreated (US), and treated catheters (TS). The NC, US, and TS showed a cell mortality < 30% at the tested dilutions (no cytotoxic effects) while a cell mortality > 30% was pointed out in PC at all the tested dilutions (cytotoxic effects).

Untreated cells were used as a blank; cells treated with the elution of an irritant reference material (latex) served as a positive control, and cells treated with the elution of a non-irritant reference material served as a negative control. Following the 24 h incubation period, cell viability was assessed using the cytotoxicity through the 3-[4,5-dimethylthiazol-2-yl]-2,5 diphenyl tetrazolium bromide (MTT) assay (Thermo-Fisher, Monza, Italy) (Figure 1). After removing the culture medium, the cells were incubated for 2 h with 100 μL/well of 1 mg/mL MTT solution at 37 °C. Then, the solution was replaced with 200 μL/well of isopropanol and incubated for an additional 30 min at room temperature under medium-speed shaking. The absorbance at 570 nm was measured with a microplate reader (Tecan Infinite 200Pro model) (Tecan Infinite, Männedorf, Switzerland), deducting background. Cytotoxic assay was determined by measuring cellular viability (%) as follows:

$$\mathrm{cell}\;\mathrm{viability}\;\left(\%\right)=\frac{\mathrm{OD}\;\left(570\;\mathrm{nm}-650\;\mathrm{nm}\right)\;\mathrm{test}\;\mathrm{product}}{\mathrm{OD}\;\left(570\;\mathrm{nm}-650\;\mathrm{nm}\right)\;\mathrm{blank}}\times 100$$

The following steps are used to assess the validity of the test:

1. The mean OD value of six replicates of the blank should be ≥0.2% with a standard deviation ≤ 18%. Additionally, the media of two blank columns should not differ by more than 15%.
2. The positive control (PC) should exhibit cytotoxicity in at least the first four tested dilutions, with a standard deviation ≤ 18%.
3. The negative control (NC) should not induce cytotoxic effects in all tested dilutions and its standard deviation should be ≤18%.
4. The standard deviation of the samples should be ≤18%.

A reduction in cell viability exceeding 30% was indicative of cytotoxicity.

The calculating of the investigation ratio (IR) was performed by using Microsoft Excel version 2412, using the following formula:

$$IR=\frac{catheters\;\left(functionalized\;or\;not\right)\;cell\;viability}{negative\;controls\;cell\;viability}\times 100$$

**Figure 2:**
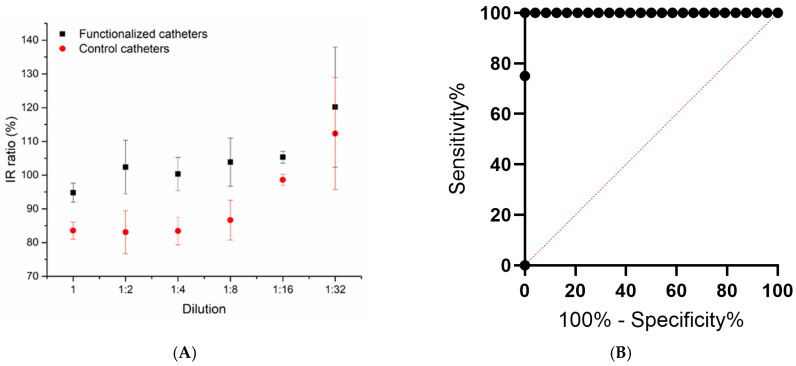
The graphs report the investigation ratio (**A**) and the ROC curve analysis (**B**).

The IR index measurement examined in this experiment allows for the evaluation of cell viability in comparison to the negative control, assessing both functionalized and non-functionalized catheters. An IR value equal to or higher than 100% relative to the negative control demonstrates a positive impact on cell behavior, while an IR value below 100% suggests an increased cytotoxicity. As illustrated in Figure 2A, the functionalized catheters demonstrated IR values close to or above 100% across all dilutions, confirming that the functionalization process does not induce cytotoxic effects. Similarly, untreated catheters also met safety standards, even though their IR values were slightly lower. This outcome may be attributed to indirect effects, such as non-specific interactions between the catheters and cells. These results remain within the safety threshold for biological applications and are not concerning.

An ROC curve analysis was performed using GraphPad Prism (version 10) to determine whether the functionalized catheter induces cytotoxic effects, similar to the positive control, or remains safe, comparable to the negative control and standard catheters. This evaluation assessed the functionalized catheter’s ability to differentiate between two categories: low cell viability (cytotoxicity) and high cell viability (safety). For the ROC curve calculation, numerical data on cell viability were provided for all samples, including positive and negative controls as well as control and functionalized catheters. These samples were assigned binary codes, with ‘1’ representing cytotoxicity (low viability) and ‘0’ indicating safety (high viability), enabling a clear distinction between the two conditions. The resulting ROC curve, displayed in Figure 2B, revealed an area under the curve (AUC) of 1.0. This value indicates perfect discrimination between cytotoxic and safe conditions within the dataset. An AUC of 1.0 demonstrates the test’s exceptional reliability in distinguishing between the two states, highlighting the functionalized catheter’s ability to either maintain cell safety or induce cytotoxic effects with high precision.

Finally, we used a multinomial logistic regression model in R (version 4.4.2) to understand which factors influence the classification of cell viability into three categories: Low, Medium, and High. Cell viability was measured in 4 samples: a positive control, a negative control, a control catheter, and a functionalized catheter. To categorize cell viability, we defined the following thresholds: values below 30% were classified as “Low”, values between 30% and 70% as “Medium”, and values above 70% as “High”. In terms of model performance, results indicate that the model successfully classified the samples into the “Low” and “High” categories. Specifically, 6 samples were correctly classified as “Low” as they belong to the positive controls associated with cytotoxic effects. While 18 samples relative to the functionalized catheter, negative control, and untreated catheter have been classified as “High”. However, the “Medium” category was never predicted due to a lack of data for this category, which was not sufficiently represented in our samples.

### 2.2. Functionalization Procedure of Silicone Catheters with Antimicrobial Rilk1-Cat

A 3 French SIL 037 catheter measuring 0.5 mm (inner diameter) × 0.99 mm (outer diameter) (Braintree Scientific, Pembroke, MA, USA) was used [19]. Catheters were cut to a length of 0.5 cm by a sterile scissor and exposed to atmospheric air plasma by using an Openair^®^ plasma generator, digital 1 KVA. The activation procedure was conducted using a power setting of 444 W, alongside a flow rate of 35 L/min and a pressure set at 86 mbar. The distance between the catheter and the plasma nozzle was set at 80 mm while moving at a rate of 40 mm s^−1^. Subsequently, the treated catheters were embedded into a Rilk1-cat peptide solution (3 mL, 0.15 μmol in 10 mM Tris-HCl, pH 8.5) for 24 h at room temperature under shaking. Afterward, the functionalized catheters were rinsed in deionized water three times to remove any unreacted peptide molecules and then air-dried at room temperature.

The stability of the functionalized material was evaluated by monitoring the potential release of the Rilk1-cat peptide in extraction solutions, following the UNI EN ISO 10993-12 standard. The tests were conducted by immersing the functionalized catheter for 24 h at 37 °C in two different extraction solutions: distilled water and a mixture of ethanol and saline (1:1). The amount of peptide released was then quantified using HPLC-UV chromatography. The results showed no detectable peptide release under the tested conditions, confirming that the peptide was strongly anchored to the catheter, exhibiting excellent stability.

### 2.3. Animal Selection, Randomization, and Sampling

Fischer 344 rats (Charles River, Calco, LC, Italy) (*n* = 44), both males and females (*n* = 22 each sex), aged between 6 and 8 weeks and weighing between 130 and 180 g, were used in the study and were compliant with the ARRIVE guidelines to fulfill the most modern and effective ethical approaches guidelines (Table S1). In particular, by thoroughly reporting all the aspects of our animal study as required by both the ARRIVE checklists, namely the 10 Essential and the Recommended set, we aimed to improve the reproducibility, and therefore the translatability, of our protocol in compliance with the best practice recommended by the National Centre for the Replacement, Refinement and Reduction of Animals in Research (NC3Rs, https://www.nc3rs.org.uk/ accessed on 24 June 2024). Weight has been used as a priori inclusion/exclusion criterion; animals weighing < 130 g have been excluded from the experiments. Sprague-Dawley rats (Charles River, Calco, LC, Italy) (*n* = 12), both males and females (*n* = 6 each sex) of the same age, were blood sampled as further control of hematological and biochemical parameters. Therefore, a total of 56 animals were used. No animals were excluded during the study. Sample size has been determined by G*Power software (3.1.9.2 version) (Universitat Dusseldorf, Germany). A Wilcoxon signed-rank non-parametric test (matched pairs) was used setting an effect size of 0.5, an α of 0.5 and a 1-β of 0.80. The resulting sample size was 10 (5 males and 5 females). However, we increased the number by one unit for each group considering the potential mortality due to adverse effects related to anesthesia, subjective response to catheterization, and achievement of the humanitarian endpoint.

Upon arrival, animals were acclimatized to the laboratory environment for 20 days. Rats were housed in a conventional facility provided with a standard pellet ration, clean drinking water, and a daily cycle of 12 h of light and 12 h of dark environments. Experimental protocols were approved on 16 June 2021 by the Italian Ministry of Health (approval No. 417/2021-PR) in compliance with the Legislative Decree 26/2014, transposing the 2010/63/EU Directive. At the beginning of the experimental trial, 2 animals/cages were housed to allow acclimatization and reduce the effects of environmental stress. Before starting the experimental trials, rats of both sexes were randomly divided into two groups, T7 and T14, each divided into 2 subgroups (*n* = 11), control (No-AMPs) and treated (AMPs). Animals have been randomly assigned to the different groups using the online GraphPad tool by Dotmatics (https://www.graphpad.com/quickcalcs/randomize1/, accessed on 20 January 2025) (Boston, MA, USA). After randomization, rats were singly housed until the experimental endpoint. Each cage has been identified by identification tags. Environmental enrichment was guaranteed by equipping each cage with nesting paper and tunnels.

**Figure 3:**
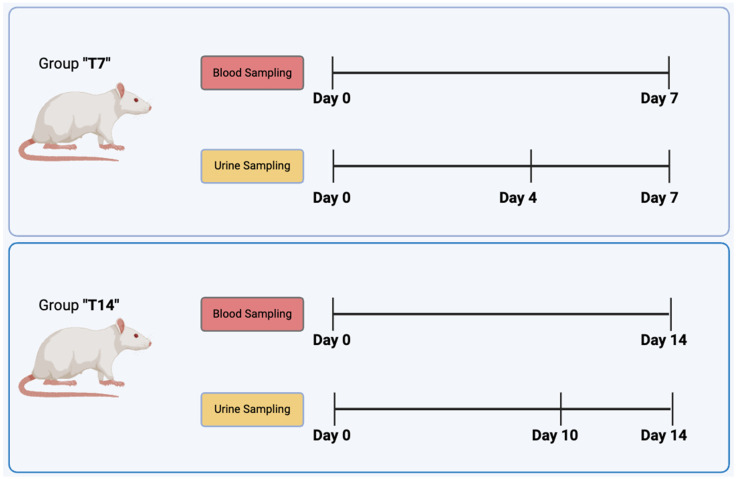
Experimental design: male and female rats were divided into two groups, T7 and T14, and further sub-divided into two more groups, No-AMPs (control) and AMPs. Blood and urine samplings were performed at the indicated time points.

According to the experimental design, reported in Figure 3 urine sampling was set at defined time points (days 0, 4, and 7 and 0, 10, and 14 for groups T7 and T14, respectively), while blood sampling was performed before surgery and at the experimental endpoint (days 0 and 7 and 0 and 14 for groups T7 and T14, respectively).

Animal care keepers, the surgeon, technicians, and outsourcing laboratory operators were aware of the group allocation at the different stages of the experiment.

At the defined time point, all animals were deeply anesthetized (ketamine 60 mg/kg i.p. + 0.3. mg/kg medetomidine) and euthanized through cervical dislocation. Catheters were then explanted from the bladder, placed in sterile tubes, and sent to the laboratory for biofilm formation assessment. Bladders were harvested for histological analyses (*n* = 3 samples/rat; whole organ). The interactions of the different units composing the research team are reported as Figure S1.

### 2.4. Surgical Procedure

**Figure 4:**
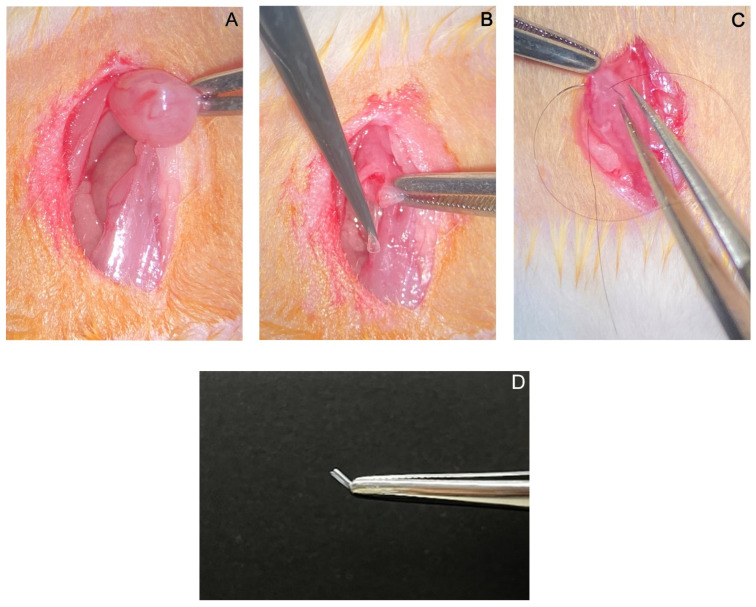
Step-by-step description of the surgical procedure: bladder exposition (**A**); catheter introduction into the bladder lumen (**B**); bladder wall suture (**C**). In (**D**) it is displayed the implanted catheter.

Rats were anesthetized (ketamine 60 mg/kg i.p. + 0.3. mg/kg medetomidine) (ACME, Cernusco sul Naviglio, MI, Italy and Orion Corporation, Turku, Finland) and placed in a supine position while the limbs were gently fixed to the operating table. Presurgical skin preparation was then performed by trichotomy of the ventral abdomen surface followed by 3 sequential scrubs of povidone–iodine (Nuova Farmec, Settimo, VR, Italy) and alcohol. Afterwards, a lower abdominal incision was made along the linea alba by a ventral midline celiotomy just cranially to the pubis, and the bladder was exposed (Figure 4A). The bladder was then isolated using gauze sponges moistened with saline solution. Organ drainage was made by cystocentesis using a fixed needle 1 mL syringe, and the urine was collected. Afterwards, a craniocaudal full-thickness bladder wall incision of a few millimeters (mm) was carefully performed on the avascular ventral surface of the bladder to avoid damaging the large blood vessels and ureters at the trigone. A sterile 5 mm catheter was introduced into the lumen using micro tweezers (Figure 4B). The bladder and the abdominal wall were then sutured with a 9/0 absorbable monofilament and a 5/0 polyfilament (Ethicon, Raritan, NJ, USA), respectively (Figure 4C). The bladder was also omentalized to promote wound healing and prevent adhesions to the adjacent structures. The abdomen was irrigated with a warm saline solution and sutured. In addition, the skin was closed with a 5/0 suture (Ethicon, Raritan, NJ, USA) with a small atraumatic needle, suturing the cystotomy site with a simple, continuing, single-layer suture, avoiding the suture material protrusion into the bladder lumen.

Animals were awakened by atipamezole administration (1 mg/kg) (Orion Corporation, Espoo, Finland). Heating pads were used during awakening. Post-surgery analgesia was guaranteed by an oral administration of paracetamol (100 mg/kg) (CEVA, Italy) in drinking water. The pain assessment was continuously performed, and the pain management protocol also included subcutaneous administration of buprenorphine (0.5 mg/kg) if required.

At the end of the procedure, all animals were singly housed and monitored to evaluate the onset of clinical signs. No antibiotics were administered.

### 2.5. Cystocentesis and Urine Analysis

Animals of both experimental groups underwent an ultrasound-guided cystocentesis (Vevo 2100 Image System, Visualsonics, Amsterdam, The Nederlands) performed under sevorane (Zoetis, Roma, Italy) administration on days 4 and 10, post-surgery, respectively. For the procedure, a 1 mL syringe equipped with a 26-gauge needle was used, and 0.2 mL of urine was collected on average at each time point. Urine was collected in 1.5 mL sterile tubes and sent to the laboratory for microbiological tests. Urine samples underwent bacteriological and mycological analyses.

### 2.6. Blood Sampling

Blood sampling was performed before surgery and 7 and 14 days after according to the experimental design (Figure 1). Withdrawals were performed from the lateral tail vein before surgery and by cardiac puncture under deep anesthesia at the experimental endpoints. At each withdrawal, 0.5 mL of whole blood was sampled using a 1 mL syringe equipped with a 26-gauge needle [20]. Of this blood quantity, 0.2 mL was immediately placed in a tube with K3 ethylenediaminetetraacetic acid (FL Medical, Torreglia, PD, Italy) asan anticoagulant for the hemachrome test and stored at 4 °C. The remaining 0.3 mL were placed into an Eppendorf tube for 10 min, then centrifuged at 3500 rpm for 15 min. The resulting sera were collected, transferred into other tubes, and stored at 4 °C. Afterwards, all the samples were sent to the laboratory for analysis. In Sprague-Dawley rats, blood sampling was performed on the day corresponding to day 0 of the Fischer rats of the groups undergoing catheter implantation.

### 2.7. Histology

Whole bladders were fixed in Bouin liquid, made of picric acid 1.2% aqueous solution (Bio-Optica, Milano, Italy), formaldehyde 37% (J.T. Baker, Amsterdam, The Netherlands), and glacial acetic acid (Carlo Erba, Milano, Italy) for 24 h, dehydrated in graded ethanol (Carlo Erba, Milano, Italy) concentrations (70% to absolute), and clarified through two baths in xylene (Bio-Optica, Milano, Italy). Afterwards, samples were embedded in paraffin wax (Bio-Optica, Milano, Italy) at 56–58 °C in a thermostat-vacuum (Jeiotech, Daejeon, Republic of Korea) for 1.5 h. Sections of 7 µm thick were obtained by using a microtome, mounted on glass slides (Labsolute, Renningen, Germany), stained with hematoxylin-eosin (HE) (Bio-Optica, Milano, Italy), and cover-slipped for morphological evaluation. Images were observed and analyzed with a Leica-DM6B (Leica, Wetzlar, Germany) and processed with LasX software, version 5.1. Digital raw images were optimized for image resolution, contrast, evenness of illumination, and background using Adobe Photoshop CC 2018 (Adobe Systems, San Jose, CA, USA).

## 3. Results

### 3.1. Housing, Post-Operative Care, and Clinical Score

The daily observation of the rats did not reveal any signs of stress or discomfort either when the animals were double- or single-housed. Cages were kept in the open air, without a filter, to allow sensory communication (visual and olfactory cues).

The daily monitoring of both the animals and the surgical area revealed that the methodology followed, in terms of surgical procedure and post-operative management, guaranteed the optimal preservation of animal health and welfare.

The “severity” assessment of the procedure has been conducted according to the methodology reported in Table 1. All the procedures included in the trial (surgery, catheter placement, cystocentesis, and blood sampling) have been predicted in detail for the most likely adverse effects setting up a specific refinement strategy for each, along with the identification of the related humane endpoints according to Smith et al. [21].

Concerning diet, the reported “supplement diet” means that standard diet and drinking water were also available for the animals, who were free to choose the preferred one based on their health status. Variations in defecation were evaluated from the second day onwards, indicating that the animal had fed and had a regular intestinal transit. The evaluation of the first feces was excluded as indicative of the pre-surgical procedure. Feces’ consistency was also checked, considering that small and dry stools correspond to a dehydration condition, while normal stools or diarrhea, instead, may be due to intestinal inflammation, potentially caused by the procedures. As for urine, the volume has been estimated based on the litter status.

Upon monitoring all these aspects, no signs of stress or discomfort were detected.

Two animals, one belonging to the 7-day group and one to the 14-day group, were found dead the day after surgery. Noteworthy, in all three cases at the necropsy, any macroscopic alterations, either of the surgical site and/or of the surrounding tissues and organs, were observed.

### 3.2. Minimally Invasive Monitoring and Urinary Bacterial Profile

The ultrasound imaging of the urinary bladder was selected as a minimally invasive procedure, alternatively to the use of metabolic cages, to collect urine in aseptic conditions and to monitor over time in situ catheter position. The procedure was performed at time points 0, 4, and 7 days after surgery and 0, 10, and 14 days after surgery on animals belonging to the T7 and T14 groups, according to the experimental design (Figure 3). Notably, at time 0, the collection of urine failed, or only an insufficient amount to conduct microbiological analyses was withdrawn.

**Figure 5:**
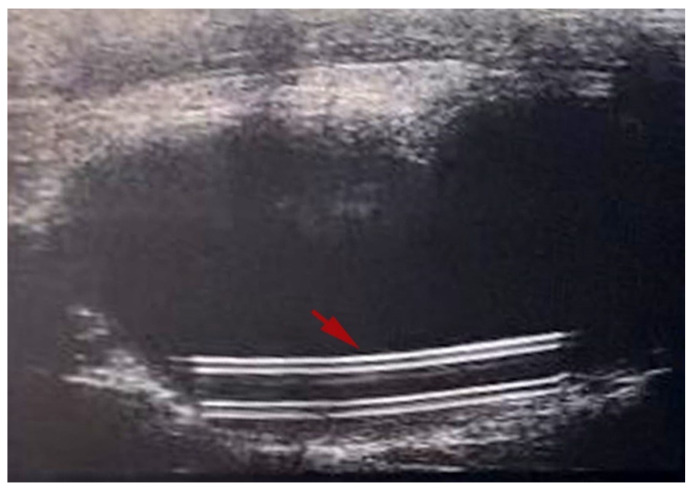
Minimally invasive monitoring made by ultrasound showing the urinary bladder at day 4. The catheter, indicated by the red arrow, appeared well positioned in situ.

The ultrasound images displayed the catheter well-positioned in situ (Figure 5) in all animals.

The microbiological status of urine in animals at 10 and 14 days in the T14 group was assessed, while at 4 and 7 days in the T7 group it was not due to the low amount of collected urines. Figure 4 displays the plot of the percentage of presence and the prevalence of the diverse bacterial strains reported by the laboratory.

**Figure 6:**
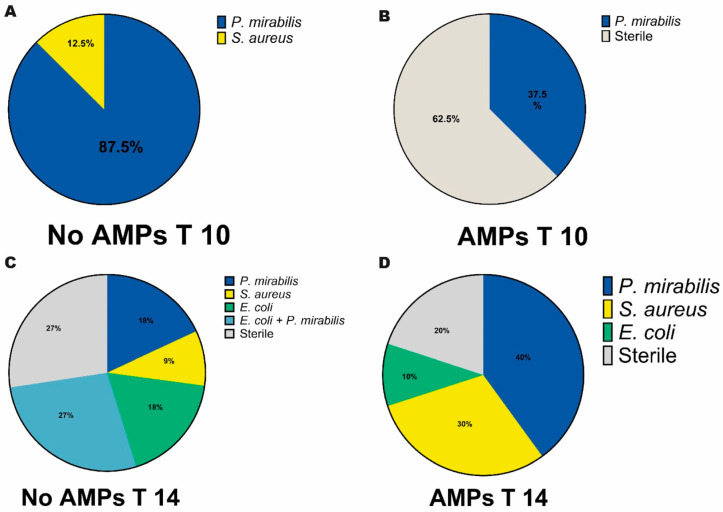
Urine bacterial profile. The graphs report the prevalence of pathogens in urine samples of No-AMPs (control) and AMPs animals at 10 days and 14 days post-surgery.

In the No-AMPs group holding the catheter for 10 days, the results of the urine culture test showed a prevalence of *Proteus mirabilis* (*P. mirabilis*) accounting for 87.5%, whereas *Staphylococcus aureus* (*S. aureus*) accounted for 22.5% (Figure 6A). In the AMPs group, 62.5% of the sample tested revealed a urine sterility while the 37.5% showed the presence of *P. mirabilis* (Figure 6B). At 14 days, in the No-AMPs group, the analyses displayed 27% of occurrence of *P. mirabilis* in association with *Escherichia coli* (*E. coli*), 18% of occurrence of only *P. mirabilis* and *E. coli*, 10% of the samples tested positive against *S. aureus*, and 27% resulted sterile (Figure 6C).

In the AMPs group, the samples analysis showed a sterile condition in 20% of cases while the presence of *P. mirabilis*, *S. aureus*, and *E. coli* was detected in 40%, 30%, and 10% of animals, respectively (Figure 6D). The values of colony-forming units per ml ranged from 10^3^ to ≤10^6^ for all the strains in all the samples analyzed. All the samples tested negative for mycological analysis.

### 3.3. Hematological and Biochemical Profiles

Hematological parameters were evaluated to monitor the general health status of the animals and to justify potential macrohematuria and microhematuria. These two conditions indeed may be caused by acute inflammation of the luminal side of bladder mucosa and by mechanical damage induced by catheter placement. Kidney function parameters were assessed to highlight the occurrence of ascending infections due to the presence of the device and to the surgical procedure.

The results of both hemachrome and kidney function parameters are reported in Table 2 as average values recorded in animals belonging to control and treated groups at 7 and 14 days post-surgery, along with species reference values. Data analysis confirmed that parameters were within ranges except for the group of males in the AMPs group at T7, displaying a low number of red blood cells and low levels of hemoglobin. Less critical anemia was observed in male and female control animals of the T14 group. However, these values were rescued at the end of the experimental trials, suggesting that the animal management was good to ensure the animal health status. Higher values of calcium were observed in all animals, independently of the group and/or the experimental time points. To exclude that these values were mainly due to strain-specific differences, as the hematological range values used referred to the Sprague-Dawley strain, those ranges were compared with the blood samples obtained by a control group of age-matched Sprague-Dawley rats maintained under the same husbandry conditions. The Table S2 displays the values of the hematological and biochemical parameters that are comparable to those detected in the Fischer 344 rats.

### 3.4. Anatomical Observations

At the end of the trial, histological analyses of the bladder tissue revealed a well-preserved anatomical structure.

**Figure 7:**
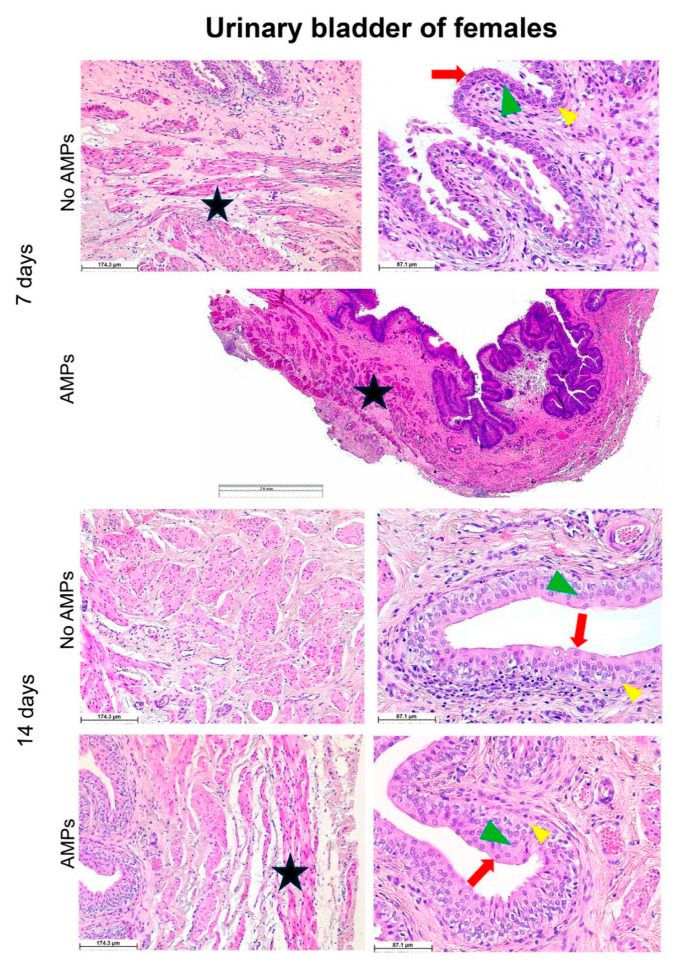
Histological analysis of the urinary bladder of males. Hematoxylin–eosin staining reveals a well-preserved anatomical structure of the urothelium and muscle layer at both endpoints, 7 and 14 days post-surgery. Red arrows, green arrowheads, and yellow arrowheads indicate umbrella, intermediate, and basal cells, respectively, while the star indicates the muscle layer. In the image representative of the No AMPs 14 days group, the muscle tissue is clearly visible throughout the picture interspersed within the connective. The value of the scale bar in the figure AMPs in the 7 days group is 1.6 mm.

**Figure 8:**
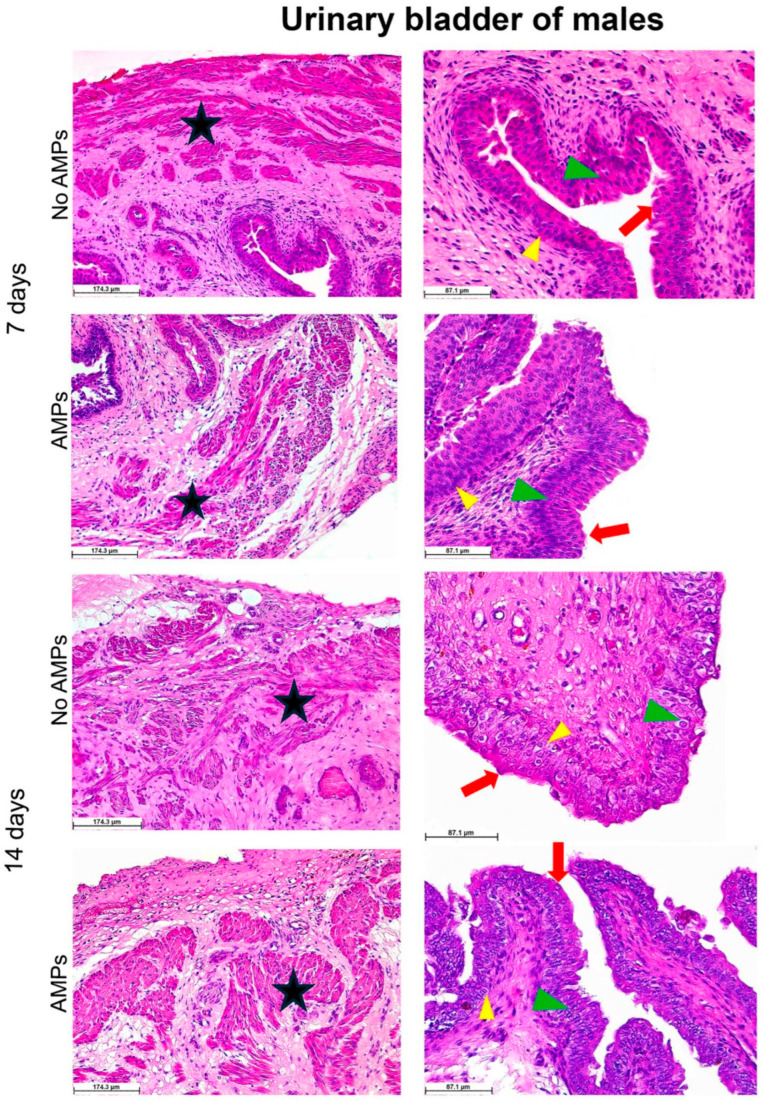
Histological analysis of the urinary bladder of females. Hematoxylin–eosin staining reveals a well-preserved anatomical structure of the urothelium and muscle layer at both endpoints, 7 and 14 days post-surgery. Red arrows, green arrowheads, and yellow arrowheads indicate umbrella, intermediate, and basal cells, respectively, while the star indicates the muscle layer.

More in detail, the preservation of the mucosa morphological features confirms the preserved ability of the organ to stretch according to its physiological condition. The urothelium clearly displays its distinctive characteristics of highly specialized epithelium acting as a permeability barrier. The phenotypic changes occurring throughout the different mucosal layers consisting of three cell types, namely basal, intermediate, and umbrella, are easily distinguishable in both male (Figure 7) and female (Figure 8) animals. In particular, no signs of inflammatory infiltration were observed in the analyzed tissues, meaning that neither the migration of macrophages and/or lymphocytes into the urothelium nor any fibrotic process infiltrating one or more of the three layers that form the mesh network of the detrusor muscle were detected.

## 4. Discussion

More than 19,000 catheter-associated urinary tract infections occurred nationwide in the USA in 2019, with attributable costs well over USD 1000 per CAUTI [22]. The high incidence of antimicrobial resistance makes CAUTI clinical management a complex issue [7]. More precisely, 80% of all UTIs without primary structural alterations are caused by *Escherichia coli* (*E. coli*) [23]. In an extensive review by Ku and coworkers, it has been recently reported that an increased complexity currently features UTI management due to the high presence of multidrug-resistant (resistance to ≥3 antibiotic classes) uropathogenic *E. coli* [24].

Applied anatomy plays a key role in the advancement of medical knowledge by coupling anatomical research and medical sciences. An effective synthesis of the relationship between anatomy and clinic is that stated by the Austrian anatomist Werner Platzer, who said, “Anatomy without clinic is dead, clinic without anatomy is deadly” [25,26], emphasizing the central role of anatomy also as a bridge towards new horizons in clinical discovery, in particular for surgically related specialties [27].

The appropriate knowledge of surgical anatomy along with the refinement of husbandry and peri-operative care is also essential for reducing animal suffering [28]. In line with this principle, and compliant with data-driven preoperative planning based on anatomical data as well as the ARRIVE guidelines [12], in our experimental design we have a priori identified appropriate actions, such as the identification of adverse effects and of commensurate humane endpoints as well as the improvement of surgical and husbandry techniques, including the adequate environmental enrichment.

Preclinical trials, including surgical procedures related to the insertion of catheters into the bladder, can cause ascending infections suitable to evolve into pyelonephritis and, in the most severe events, into septicaemia in case of inappropriate device handling and sterility conditions. Therefore, the onset of any ascending infections should be minimized by pre-operative antibiotic therapy. This latter, on the other hand, may interfere with data interpretation and with the overall output of the study in the case of research aimed at establishing the effectiveness of a specific device functionalized to hold intrinsic antimicrobial properties. In this scenario, we set up a fast and safe surgical procedure, avoiding the use of antibiotics while allowing observation for a relatively prolonged period of time (2 weeks). Compared to what generally happens when transurethral placement or tunneling is performed, we did not observe hematuria, inflammatory infiltration, or any kind of distress; therefore, we can confirm that our model preserves animal well-being. The control groups were indeed expected to suffer a higher severity due to the absence of the supposed protective effects of the antimicrobial peptides coating the catheter. Noteworthy, the only medications included in the protocol, besides anesthetics, were paracetamol, administered for the first 24 h post-surgery to all the animals, and buprenorphine, never administered as none of the animals showed signs of pain. According to the experimental design, blood sampling was performed before surgery and 7 and 14 days after it, while ultrasound-guided cystocentesis was performed under sevorane administration at days 4 and 10, post-surgery, respectively. Therefore, we can sustain that the medications did not interfere with the results of the analyses. In addition, the rigorous respect of aseptic conditions in the pre- and post-surgery phases helped to control and limit the spread of secondary infections in the urinary tract, including the bladder. The colony-forming units of pathogens detected in the urine were not sufficient to determine a manifested clinical infection. Morphological observations of the bladder wall, displaying a totally preserved anatomical structure in the absence of lymphocytic infiltration, further corroborate this observation (Figure 7 and Figure 8).

Noteworthy, with the aim of reaching the experimental goal while respecting animal welfare, we relied on a minimally invasive imaging procedure, such as ultrasound-guided cystocentesis (Figure 5). On the other hand, as described in a very recent work by Mihai et al., urology has recently reached new frontiers using laparoscopic intraoperative ultrasound. This technique, by allowing high-quality visualization of internal organs, ensures a constant monitoring of the surgical procedure in real-time, enhancing its adequacy and avoiding vascular injuries and superabundant removal of healthy tissue [29]. In the present study, the use of an ultrasound-guided approach allowed us to avoid the use of metabolic cages even for 24 h, corresponding to mild severity according to the Directive 2010/63/EU, with well-established detrimental effects on rodents welfare [30,31], and to assess the anatomical integrity of the urinary bladder as well as the in situ position of the medical device. Remarkably, the ultrasonography enabled us to sample urine under aseptic conditions, necessary to evaluate the efficacy of functionalized catheters at intermediate time points.

As for the husbandry aspects, with respect to the species-specific ethology, single-housed animals were exposed to visual, auditory, and olfactory contacts with conspecifics along the whole experimental period [32]. Therefore, we recognize some limitations of the study related to the relatively small sample size and the difficulty in obtaining sufficient urine volume at the established time points; however, we decided to incur these biases in favor of the principles of Reduction and Refinement by using the smallest possible number of animals and cystocentesis instead of metabolic cages for urine sampling. Finally we hypothesize that the proposed refined protocol underlies the preservation of tissue microarchitecture and whole organ integrity, maintaining the most specific feature of the urothelium, namely, its capacity to rearrange the thickness of its wall depending on the stretching levels [33]. As modern approaches to diagnosis and therapy allow effective treatments of several diseases, highlighting the need for multidisciplinary strategies, the role of anatomical science becomes even more crucial to contribute to facing ongoing challenges in clinical practice.

## 5. Conclusions

With this report we have demonstrated how the development and appropriateness of surgical models in the translational research rely on a combination of several aspects, ranging from the anatomical knowledge to surgical techniques and husbandry management, all aspects that are necessary to alleviate animal suffering and move forward in translating basic science findings into health-care advances.

## Figures and Tables

**Table 1:** The table reports the methodology followed to assess the severity of the different procedures performed by considering the potential adverse effects of each along with the refinement strategy and the humane endpoints applied.

Procedure	Potential Adverse Effects	Refinement Strategy	Humane Endpoint
Surgery	Body weight decrease	Softened food directly available at the bottom of the cage	>10% weight decrease
		Diet supplements (i.e., gel diet, sunflower seeds)	
	Kyphosis/Antalgic attitude	Analgesic drug administration	Analgesic drug inefficacy
	Dehydration	Softened food directly available at the bottom of the cage	
		Diet supplements (i.e., gel diet)	
		Supplemented drinking water (for ex. with protein powder, vitamins, minerals)	
		Supplemental parental administration of saline solution (intraperitoneal, subcutaneous)	
	Ruffled/matted fur and piloerection	Supplemented drinking water (for ex. protein powder, vitamins, minerals)	
	Altered movement/gait	Softened food directly available at the bottom of the cage	Hypomobility > 24 h
	Reaction to manipulation	Tunnel handling	
	Nesting behaviour	Environmental enrichment (i.e., sizzled nest paper)	
Device placement	Anuria due to acute inflammation	Antinflammatory, analgesic drug administration	Anuria > 18 h
		Supplemental parental administration of saline solution (intraperitoneal, subcutaneous)	
	Urethra obstruction due to catheter displacement	Catheter replacement	
Cystocentesis	Urine leakage	Increased monitoring	>8 h urine leaking from the suture
	Emathuria (vascular damage)	Increased monitoring	>48 h hematuria
Blood sampling	Reaction to manipulation	Tunnel handling	
	Hematoma formation	Analgesic ointment application	

**Table 2:** The table reports the average values of the hematological and biochemical parameters of the animals included in the study.

		CTRL				TREATED			
		T 0		T 7		T 0		T 7	
**Haemochrome**	**Ref. values**	**F**	**M**	**F**	**M**	**F**	**M**	**F**	**M**
RBC M/μL	7.00–11.00	6.17	8.07	7.59	7.47	7.22	7.57	7.53	5.58
Hgb g/dL	10.0–20.0	10.7	14.0	13.2	12.2	12.6	9.9	11.8	8.8
HCT %	35.0–40.0	32.5	41.2	38.7	37.2	39.0	29.7	35.5	27.6
WBC m/μL	3.2–12.7	3.1	5.3	6.2	5.9	3.0	4.4	6.9	6.2
Neutr. m/μL	0.50–2.60	0.1	0.2	0.6	0.2	0.5	0.2	0.2	0.7
Lymph m/μL	0.34–9.20	2.6	4.6	5.4	5.4	2.2	3.9	6.4	5.0
PLT m/μL	190–1000	386	746	701	698	707	713	657	484
**Kidney function**									
Urea (mg/dL)	19–27	22	22	17	16	21	21	18	18
Crea (mg/dL)	0.30–1.00	0.49	0.44	0.41	0.39	0.48	0.52	0.54	0.88
CaLcium (mg/dL)	3.20–8.00	11.2	10.93	10.88	10.8	11.2	11.47	10.6	10.54
Phosphorus (mg/dL)	6.00–10.40	7.3	7	7.13	9.8	6.8	7.07	7.4	9.82
		**CTRL**				**TREATED**			
		**T 0**		**T 14**		**T 0**		**T 14**	
**Haemochrome**	**Ref. values**	**F**	**M**	**F**	**M**	**F**	**M**	**F**	**M**
RBC M/μL	7.0–11.0	6.17	6.17	6.89	7.71	7.22	5.57	7.7	7.82
Hgb g/dL	10.0–20.0	10.7	10.7	12.5	13.9	12.6	9.9	14.3	14.1
HCT %	35.0–40.0	32.5	32.5	39.8	43.0	39.0	29.7	45.1	45.1
WBC m/μL	3.2–12.7	3.1	3.1	3.7	4.9	3.0	4.4	3.6	4.6
Neutr. m/μL	0.50–2.60	0.1	0.1	0.3	0.5	0.5	0.2	0.4	0.9
Lymph m/μL	0.34–9.20	2.6	2.6	2.9	3.9	2.2	3.9	2.7	3.0
PLT m/μL	190–1000	387	386	619	668	707	713	640	644
**Kidney function**									
Urea (mg/dL)	19–27	47	38	39	38	44	41	38	37
Crea (mg/dL)	0.30–1.00	0.49	0.44	0.40	0.37	0.48	0.52	0.33	0.37
CaLcium (mg/dL)	3.20–8.00	11.20	10.93	10.8	10.6	11.2	11.47	10.65	10.6
Phosphorus (mg/dL)	6.00–10.40	7.30	7	7.3	7	5.8	7.07	8.25	6.9

## Data Availability

The data that support the findings of this study are available on request. They are not publicly available due to privacy or ethical restrictions.

## Supplementary Materials

The following supporting information can be downloaded at https://www.mdpi.com/article/10.3390/biomedicines13020285/s1: Figure S1: The Figure reports the interactions of the different units composing the research team); Table S1: The table reports in details in which sections of the manuscript are reported all the aspects concerning the pre-clinical trial according to the ARRIVE guidelines; Table S2: The table displays the average values of the haematological and biochemical parameters of the Sprague-Dawley rats.

## Abbreviations

CAUTIs, catheter-associated urinary tract infections; UTIs, urinary tract infections; AMPs, antimicrobial peptides; *E. coli*, *Escherichia coli*; ARRIVE, Animal Research: Reporting of In Vivo Experiments; HE, hematoxylin–eosin; *P. mirabilis*, *Proteus mirabilis*; *S.aureus*, *Staphilococcus aureus*; MTT, 3-[4,5-dimethylthiazol-2-yl]-2,5 diphenyl tetrazolium bromide.
