# Supplementary material for: Well-Preserved Urinary Bladder Anatomy in Rats After Minimally Invasive Surgery
Source: Biomedicines. 2025 Jan 24;13(2):285. doi: 10.3390/biomedicines13020285 (PMC11853525; doi:10.3390/biomedicines13020285)

Well-preserved urinary bladder anatomy in rats after minimally invasive surgery.

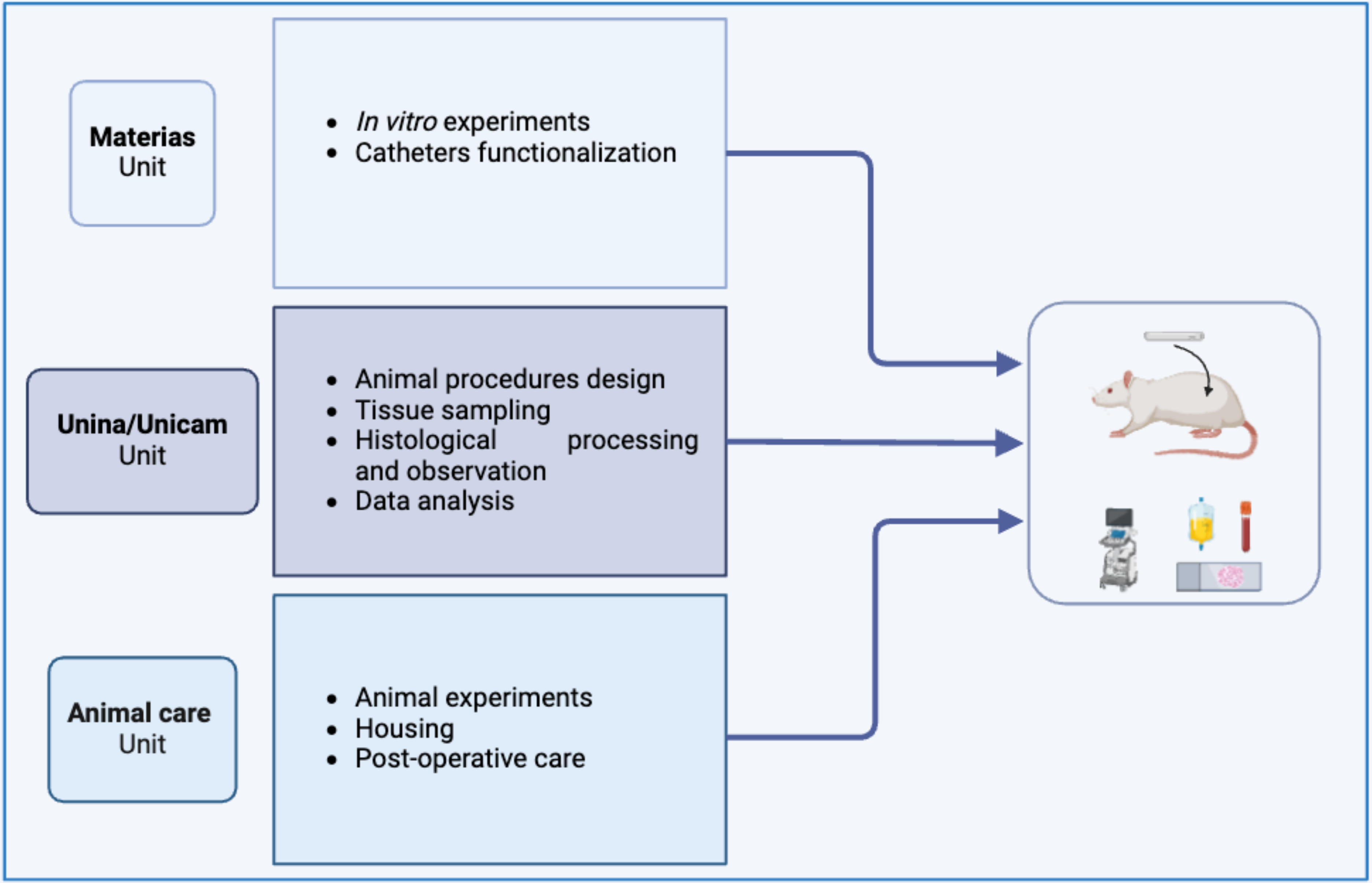

Supplement: Supplementary file 1 [file biomedicines-13-00285-s001.zip › Supplementary/Figure S1.pdf]
